# Sequential Collinear Photofragmentation and Atomic Absorption Spectroscopy for Online Laser Monitoring of Triatomic Metal Species

**DOI:** 10.3390/s20020533

**Published:** 2020-01-18

**Authors:** Jan Viljanen, Kim Kalmankoski, Victor Contreras, Jaakko K. Sarin, Tapio Sorvajärvi, Hanna Kinnunen, Sonja Enestam, Juha Toivonen

**Affiliations:** 1 Photonics Laboratory, Physics Unit, Tampere University, Post Office Box 692, FI-33101 Tampere, Finland; kim.patokoski@tuni.fi (K.K.); jaakko.sarin@uef.fi (J.K.S.); tapio.sorvajarvi@mil.fi (T.S.); juha.toivonen@tuni.fi (J.T.); 2 Instituto de Ciencias Físicas, Universidad Nacional Autónoma de México, Cuernavaca 62210, Mexico; victor@icf.unam.mx; 3 Diagnostic Imaging Center, Kuopio University Hospital, Post Office Box 100, FI-70029 Kuopio, Finland; 4 Valmet Technologies Oy, Lentokentänkatu 11, Post Office Box 109, FI-33101 Tampere, Finland; hanna.kinnunen@valmet.com (H.K.); sonja.enestam@valmet.com (S.E.)

**Keywords:** photofragmentation, absorption, lead, kinetics, combustion

## Abstract

Industrial chemical processes are struggling with adverse effects, such as corrosion and deposition, caused by gaseous alkali and heavy metal species. Mitigation of these problems requires novel monitoring concepts that provide information on gas-phase chemistry. However, selective optical online monitoring of the most problematic diatomic and triatomic species is challenging due to overlapping spectral features. In this work, a selective, all-optical, in situ gas-phase monitoring technique for triatomic molecules containing metallic atoms was developed and demonstrated with detection of PbCl_2_. Sequential collinear photofragmentation and atomic absorption spectroscopy (CPFAAS) enables determination of the triatomic PbCl_2_ concentration through detection of released Pb atoms after two consecutive photofragmentation processes. Absorption cross-sections of PbCl_2_, PbCl, and Pb were determined experimentally in a laboratory-scale reactor to enable calibration-free quantitative determination of the precursor molecule concentration in an arbitrary environment. Limit of detection for PbCl_2_ in the laboratory reactor was determined to be 0.25 ppm. Furthermore, the method was introduced for in situ monitoring of PbCl_2_ concentration in a 120 MW_th_ power plant using demolition wood as its main fuel. In addition to industrial applications, the method can provide information on chemical reaction kinetics of the intermediate species that can be utilized in reaction simulations.

## 1. Introduction

Laser spectroscopy enables sensitive and molecule-specific online monitoring of gas-phase components, which can be performed in situ even in harsh process environments. A number of laser techniques have been introduced for in situ process gas analysis [1], including tunable diode laser absorption spectroscopy (TDLAS) [2], laser-induced breakdown spectroscopy (LIBS) [3], laser-induced fluorescence (LIF) [4], and direct ultraviolet (UV) absorption spectroscopy [5]. Recently, gaseous diatomic and triatomic molecules containing alkali metals and heavy metals have become an important application for online process monitoring as they have been found to cause drastic operational problems, such as corrosion and deposit formation in high-temperature combustion processes [6,7,8]. Diatomic and triatomic metallic species are challenging to detect using direct infrared (IR) absorption measurement. Typically, the characteristic vibrational transitions of these molecules are in wavelengths longer than 30 μm, making them difficult to detect with current IR technologies [9]. Moreover, in general, diatomic and triatomic molecules have broad absorption bands in UV and visible regions which tend to overlap [10]. Therefore, in an environment with multiple polyatomic species present in gas phase, it is difficult, if not impossible, to distinguish a single type of molecule.

Photofragmentation-based techniques have exhibited great potential for metal species monitoring in high-temperature processes [1]. Excimer laser-induced fragmentation fluorescence (ELIF) spectroscopy has been introduced for monitoring of alkali compounds and lead vapors in power plant boilers [11] and in combustion flames [12,13]. However, the technique struggles to identify the precursor of emitting photodissociation products induced by the laser pulse. The dissociation with wavelength of 193 nm, which is commonly used in ELIF applications, is not selective in dissociation and, hence, the emitting species can be originated from different precursor molecules [13]. Moreover, this wavelength is strongly absorbed by O_2_ and other abundant compounds present in common industrial processes. Therefore, ELIF has insufficient penetration depth for full-scale applications and heterogeneous samples and, thus, is not suitable for molecule-specific in situ monitoring of process gases.

Collinear photofragmentation and atomic absorption spectroscopy (CPFAAS) [14] has overcome the problem in distinguishing between different precursor molecules by using molecule-specific fragmentation wavelengths, as demonstrated in simultaneous quantitative detection of KCl and KOH during thermal conversion of biomass in a single particle reactor [15]. The laser pulse wavelengths applied in CPFAAS measurements are typically longer than 250 nm [15]. These wavelengths are not equally prone for absorption to abundant molecules in gas phase, such as oxygen, as laser wavelength used in ELIF. Therefore, CPFAAS has longer penetration depth to complex gas mixtures than ELIF, which has enabled its application for in situ KCl monitoring in a full-scale combustion power plant [16]. However, CPFAAS has been limited to simple alkali-containing molecules that can be fragmented with single fragmentation process. More complex molecules are dissociated relying on multiphoton dissociation [17]. For example, detection of NO_X_ molecules has been performed using multiphoton dissociation [18]. Similarly, molecules containing metallic atoms can be selectively fragmented using multiple photons [19]. However, to increase the fragment yield, the multiphoton dissociation requires a laser pulse with a high peak power. The fragment yield can be increased also by targeting the resonant transitions of the precursor molecule with two, or more, sequential laser pulses with different wavelengths. Previously, sequential laser pulses in photofragmentation have been utilized to perform double-resonance photofragmentation that allows dissociation of more complex molecules [20,21,22,23,24]. Sequential laser pulses have also enabled observing radical photodissociation behavior in photofragment translational spectroscopy [25,26,27,28]. Even though the sequential laser pulse photofragmentation has so far been focusing on studying fundamental features of molecules, with effective fragment detection, it shows potential for more applied use in process industry to monitor poly-atomic compounds in gas phase.

In this work, we present sequential laser pulse CPFAAS for detection of triatomic molecules containing metal species. The method utilizes two sequential laser pulses that are absorbed by resonant transitions of the triatomic precursor molecule and the intermediate fragment. The first laser pulse dissociates the precursor molecule and the second pulse dissociates the produced intermediate molecule producing atomic fragments. The fragments are then probed with a continuous wave (CW) laser with emission wavelength tuned to correspond to an absorption line of the desired fragment. The concentration of the precursor molecule can be determined from the decreased transmission of the probe beam after the launch of the fragmenting laser pulses [29]. The sequential CPFAAS is demonstrated using PbCl_2_. PbCl_2_ is an interesting molecule for the power and heat industry utilizing waste-derived fuels due to its corrosive effect on fire-side heat exchanger surfaces [30,31,32]. Waste-derived fuels contain elevated concentrations of heavy metals, such as lead, that decrease the first melting temperatures of ash deposits on boiler surfaces increasing the risk of molten-phase corrosion leading to a drastic corrosion rate of the boiler surface material [33]. The path of heavy metals from fuel to ash deposits is not known in detail and, hence, new monitoring concepts are crucial to improve the viability of waste combustion. This work presents the theoretical background and the experiential results based on a sequential double optical fragmentation process in a triatomic molecule, in this case PbCl_2_, expanding CPFAAS’s applicability into more complex compounds. Furthermore, the method’s field applicability is demonstrated in a 120 MW_th_ circulating fluidized bed (CFB) boiler using recovered waste wood as its main fuel.

## 2. Theory of Quantitative Detection

The approach for quantitative triatomic molecule detection with CPFAAS is described in the following using PbCl_2_ as the example molecule. Two pulsed lasers, at resonant wavelengths to the absorption into PbCl_2_ and PbCl, are used to break the PbCl_2_ molecule into PbCl and subsequently the PbCl radicals into Pb* by the excitation of the dissociative electronic states of the molecules. As the last fragment is atomic lead, a narrow-linewidth laser is used to probe the temporally increased Pb* concentration in the probe volume. The decrease in the probe beam transmission is proportional to the precursor molecule concentration enabling quantitative detection as follows.

Assuming there are no energy losses due to scattering effects, the Beer–Lambert law determines the absorption of the first laser pulse in the measurement volume as

(1) $$\frac{E_{p1}}{E_{0p1}}\;=\;\exp\left(-\alpha L\right)\;=\;\exp\left(-\frac{N_{PbCl_{2}}}{V}\sigma_{PbCl_{2}}L\right)$$

where *E_p_*_1_ and *E*_0*p*1_ stand for the energy of the first fragmentation pulse at the output and input, respectively; *αL* is the absorbance due to the number of molecules *Ν_PbCl_*_2_ present in the probe volume *V* across the probe length *L*. The term *σ_PbCl_*_2_ is the absorption cross-section of PbCl_2_ at first fragmenting laser pulse wavelength. Assuming that the dissociation efficiency of PbCl_2_ equals 1, i.e., statistically each absorbed photon produces one PbCl molecule, the number of induced PbCl, *N_PbCl_*, can be estimated as

(2) $$N_{PbCl}\;=\frac{E_{0p1}-E_{p1}}{\frac{hc}{\lambda_{p1}}}\;=\;\frac{\left(1-\frac{E_{p1}}{E_{0p1}}\right)E_{0p1}\cdot \lambda_{p1}}{hc}$$

where *λ_p_*_1_, *h*, and *c*, represent the laser wavelength of the first fragmentation pulse, the Planck’s constant, and the velocity of light, respectively. Hence, applying Equation (1)

(3) $$N_{PbCl\;}=\;\frac{\left[1-\exp\left(-\frac{N_{PbCl_{2}}}{V}\sigma_{PbCl_{2}}L\right)\right]\cdot E_{0p1}\cdot \lambda_{p1}}{hc},$$

the second laser pulse absorption to PbCl is defined by the Beer–Lambert law

(4) $$\frac{E_{p2}}{E_{0p2}}\;=\;\exp\left(-\alpha L\right)\;=\;\exp\left(-\frac{N_{PbCl}}{V_{p1}}\sigma_{PbCl}L\right)$$

where *E_p_*_2_ and *E*_0*p*2_ stand for the energy of the second fragmentation pulse at the output and input, *V_p_*_1_ represents the volume containing PbCl molecules, and *σ_PbCl_* is the absorption cross section of PbCl molecules at the wavelength of the second laser pulse. Assuming that fragmentation efficiency equals 1, the number density of dissociated Pb atoms can be estimated as

(5) $$N_{Pb\;}=\;\frac{\left[1-\exp\left(-\frac{N_{PbCl}}{V_{p1}}\sigma_{PbCl}L\right)\right]\cdot E_{0p2}\cdot \lambda_{p2}}{hc}.$$

The CW probe beam absorption due to Pb atoms, released in the volume *V_p_*_1_ (*V_p_*_1_ *= V_p_*_2_) upon the second fragmentation process, can be expressed as:

(6a) $$\frac{I}{I_{0}}\;=\;\exp\left(-\alpha L_{max}\right)\;=\;\exp\left(-\frac{N_{Pb}}{V_{p2}}\sigma_{Pb}L\right),$$

where *I* and *I*_0_ represent, respectively, the transmission intensities of the probe laser before and after second laser pulse creating free Pb atom population and *αL_max_* and *σ_Pb_* are the absorbance and the absorption cross-section for Pb atoms, respectively. From Equation (6a), the number of Pb atoms, *N_Pb_*, can be estimated by measuring from transmission intensity change of the probe beam *I/I*_0_ by:

(6b) $$N_{Pb}\;=\;\frac{-\ln\left(\frac{I}{I_{0}}\right)\cdot V_{p2}}{\sigma_{Pb}L}.$$

As the second fragmenting laser pulse is launched shortly after the first fragmenting laser pulse, i.e., no loss of PbCl occurs between the pulses, an expression to determine PbCl_2_ concentration can be obtained using Equations (2), (5), and (6b):

(7) $$x_{PbCl_{2}}\;=\;-\ln\left\{1+\ln\left[1+\ln\left(\frac{I}{I_{0}}\right)\frac{A_{p2}}{\sigma_{Pb}E_{0p2}}\frac{hc}{\lambda_{p2}}\right]\frac{A_{p1}}{\sigma_{PbCl}E_{0p1}}\frac{hc}{\lambda_{p1}}\right\}\frac{kT}{p}\frac{1}{\sigma_{PbCl_{2}}L},$$

where *A_p_*_1_ and *A_p_*_2_ are the cross-sectional areas of the first and second fragmenting laser beams, respectively. To obtain Equation (7), the ideal gas law is applied and the terms *k*, *T*, and *p* represent the Boltzmann constant, and temperature and pressure at measurement volume, respectively. Thus, Equation (7) can be used to calculate the gas-phase concentration of PbCl_2_ applying the decrease in probe beam transmission intensity due to induced atomic Pb population in the gas phase.

The population of induced Pb in the probe beam volume increases only temporally after the sequential photodissociation at time *t* = 0. The transmission of the probe beam returns to its original level due to relaxation and recombination processes of produced absorbing fragments. Assuming an exponential decay of absorption, CPFAAS temporal evolution can be obtained by Beer–Lambert law according to [29]:

(8) I(t) ={I0, t<0I0exp(−aLmaxexp(−tτ)), t≥0 .

Here, *τ* represents the 1/e decay time constant for the decay process.

In case of an impure sample in turbid media typical to industrial processes, in addition to absorption to PbCl_2_ molecules, fragmenting laser pulse energies attenuate due to absorption to other species and scattering. To compute the PbCl_2_ concentration in such conditions, the additional extinction of the laser beams has to be considered. Therefore, Equations (1) and (4) have to be rewritten as

(9) $$\frac{E_{p}}{E_{0p}}=\;\exp\left(-\alpha L\right)\exp\left(-\beta L\right)$$

where *β* is the attenuation coefficient of a fragmenting laser pulse by the scattering and absorption due to other species. Because of additional extinction, differential calculus has to be used in the derivation of concentration. Unfortunately, the general analytical expression cannot be derived. For practical purposes, an approximation can be used that the attenuation of the second pulse is mainly due to extinction (*α_PbCl_ << β_p_*_2_). This is well justified, for example, with full-scale power plant boiler measurement results. Typically, in a boiler environment, the extinction is high due to the particle load and other absorbing species and, on the other hand, the PbCl_2_ concentration that contributes to the induced PbCl population is typically low, rarely exceeding tens of ppm. When the approximation is applied, the concentration of PbCl_2_ can be calculated as

(10) $$x_{PbCl_{2}}\;=\;\ln\left(\frac{I_{0}}{I}\right)\frac{ln\left(\frac{E_{0p1}}{E_{p1}}\frac{E_{0p2}}{E_{p2}}\right)}{\left(1-\frac{E_{p1}}{E_{0p1}}\frac{E_{p2}}{E_{0p2}}\right)}\frac{hc}{\;\lambda_{p1}E_{0p1}}\frac{hc}{\;\lambda_{p2}E_{0p2}}\frac{A_{p1}}{\sigma_{PbCl_{2}}}\frac{A_{p2}}{\sigma_{PbCl}}\frac{1}{\sigma_{Pb}L}\frac{kT}{p}$$

where *E_p_*_1_ and *E_p_*_2_ are the measured pulse energies after the sample. The high extinction of fragmenting laser pulses causes the measurement to be weighted to the first few meters of the measurement path. This may lead to measurement error in long measurement paths if the medium cannot be considered homogeneous.

Equations (7) and (10) describe how measurable quantity *aL_max_* = ln(*I*_0_*/I*), i.e., the probe beam absorbance immediately after the fragmentation laser pulses, and arrangement specific variables *E*_0*p*1_*, E*_0*p*2_*, E_p_*_1_*, E_p_*_2_*, A_p_*_1_*,* and *A_p_*_2_ can be used to determine the precursor molecule concentration. Other parameters are sample specific or physical constants. In Section 4.1, the sample specific parameters *σ_PbCl_*_2_, *σ_PbCl_*, and σ_Pb_ are determined for calibration-free monitoring of PbCl_2_ in an arbitrary environment.

## 3. Materials and Methods

**Figure 1:**
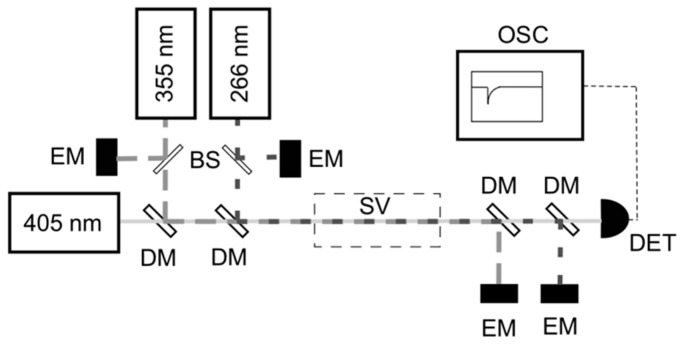
Experimental setup for the gas-phase detection of PbCl_2_. The transmission of the probe laser (405 nm) through a sample volume (SV) was monitored by a photodiode (DET) for collinear photofragmentation and atomic absorption spectroscopy (CPFAAS) signal upon double photofragmentation process of 355 nm and 266 nm laser pulses. Pulse energies were monitored by energy meters (EM) at the input and output. Beam splitters (BS) and dichroic mirrors (DM) were used to split, direct, and co-align the beams. An oscilloscope (OSC) was used for recording the signal for further analysis.

**Figure 2:**
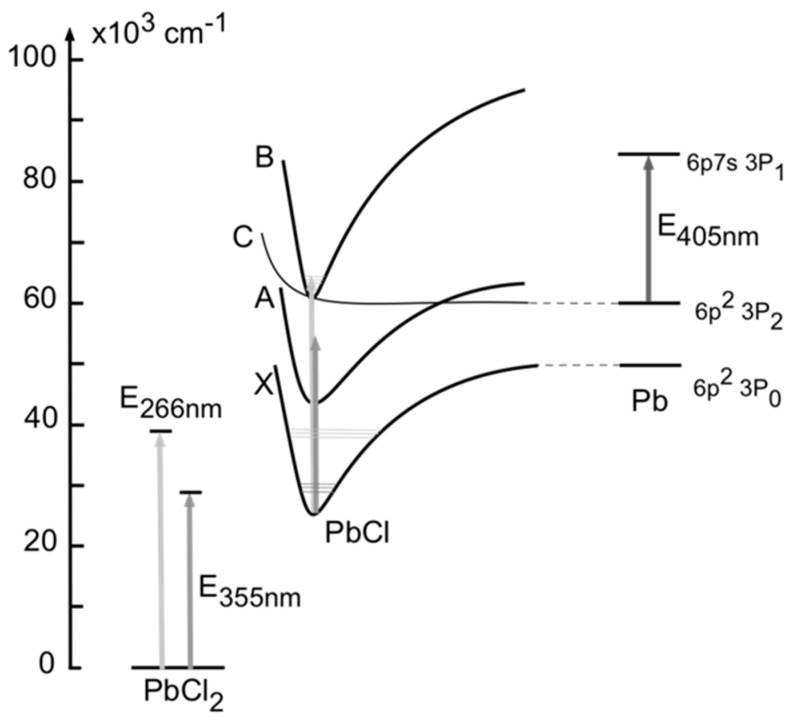
Simplified energy levels diagram for PbCl molecules and Pb atoms adapted from [35]. The Pb transition associated with the optical absorption of light at 405.79 nm involved the excited states 6p2 ^3^P_0_ and 6p7s ^3^P_1_. According to the diagram, the 6p2 ^3^P_0_ level of Pb atoms can be achieved upon a double photodissociation process of PbCl_2_ molecules using laser pulses at 355 nm and 266 nm wavelengths.

Initial sequential CPFAAS experiments with PbCl_2_ were performed in controlled laboratory conditions to validate the theoretical derivation and obtain absorption cross-section values for the PbCl_2_ fragments. The experimental arrangement for PbCl_2_ detection is presented in Figure 1, consisting of two pulsed UV lasers and a CW narrow-linewidth diode laser (Sure Lock, Ondax, Santa Clara (CA), United States of America). The first fragmentation process was induced by the third harmonic (355 nm) of an Nd:YAG pulsed laser (Ultra Big Sky series, Quantel, France) emitting 5 ns wide pulses with a repetition rate of 10 Hz. The PbCl_2_ molecules were excited to a dissociative state due to absorption of the 355 nm pulses producing PbCl + Cl fragments. The second fragmentation process of PbCl into Pb* + Cl fragments was induced by the absorption of the second laser pulse from the fourth harmonic (266 nm) of an Nd:YAG laser (FQSS-266-200, CryLas GmBh, Berlin, Germany) having a temporal width of 1 ns. The simplified energy level diagram of the fragmentation process is presented in Figure 2 demonstrating that the produced Pb atoms were in excited (*) state. Hence, the probe laser wavelength was chosen accordingly to be 405.789 nm. The probe laser output was divided into the probe and reference beams using a wedge window (BS, WG10050-A, Thorlabs, Newton (NJ), United States of America). The reference beam was directed through a lead hollow cathode lamp (Heraeus, Hanau, Germany) containing vaporized Pb* atoms. The wavelength of the CW probe laser was tuned and actively locked to the Pb* absorption profile at 405.789 nm [34] by monitoring the reference beam transmission through the lamp. The beam transmitted through BS was used as the probe beam in the CPFAAS arrangement. To verify the probe beam wavelength, it was measured with a wavelength-meter (WA-1500-VIS, EXFO Burleigh) with an accuracy of 0.1 pm. The three laser beams, two pulsed and one CW, were aligned in a collinear optical path through the sample volume. Their cross-section areas were expanded and limited to a circular aperture of 7 mm in diameter. The beams were aligned on the collinear path and separated after passing the sample by using appropriate dichroic mirrors (DM, Semrock, Rochester (NY), United States of America). Pulse energies were monitored with photodiode energy sensors (EM, PD-10, and PE-9, Ophir, Jerusalem, Israel, and two J25MB-LE, Coherent, Santa Clara (CA), United States of America) at the input and output of the sample volume. The probe laser intensity transmitted through the sample was monitored using a biased silicon photodiode (DET, DET10A, Thorlabs, Newton (NJ), United States of America) allowing a fast response in the ns-scale for CPFAAS signal detection. A digital oscilloscope (OSC, HDO6054, LeCroy, Chestnut Ridge (NY), United States of America) was used to record the signal for further analysis. Customized software was used to control the measurements from a portable computer. The software controlled and monitored the probe wavelength locking, synchronized the interpulse delay firing of the laser pulses, and the measurement of pulse energies (Pulsar-2 interface, Ophir, Jerusalem, Israel) and acquired the CPFAAS signal.

In laboratory studies, the PbCl_2_ vapor was produced by heating solid PbCl_2_. The lead (II) chloride powder (268,690, Sigma Aldrich) was contained in a quartz tube that was placed into an electrical tube oven. Optical path length through the sample volume was 0.6 m. The experiments were carried out at N_2_ or at N_2_-O_2_ atmosphere and at atmospheric pressure. The amount of PbCl_2_ at gas phase was varied during the experiment by controlling the temperature of the oven. The set temperature during experiments in constant temperature was 493 °C. To reach the desired measurement conditions, the sample tube was flushed with the premixed gas mixture for 300 s. After flushing, the sample volume was let 120 s to settle into an equilibrium before recording the desired measurement signal. It was experimentally validated that 120 s was sufficient to reach static temperature and PbCl_2_ vapor concentration above the solid PbCl_2_ sample. The equilibrium concentrations of PbCl_2_ vapor at each temperature were computed using commercial thermochemical database HSC 5.1 (Outokumpu Research). The database has been previously utilized, for example, to validate quantitative KCl measurement with CPFAAS. The KCl results were compared also with differential optical absorption spectroscopy (DOAS) showing high correspondence. [29] Therefore, HSC 5.1 database was relied on also in the case of PbCl_2_.

## 4. Results and Discussion

### 4.1. PbCl_2_, PbCl, and Pb* Absorption Cross-Sections for Quantitative Monitoring

**Figure 3:**
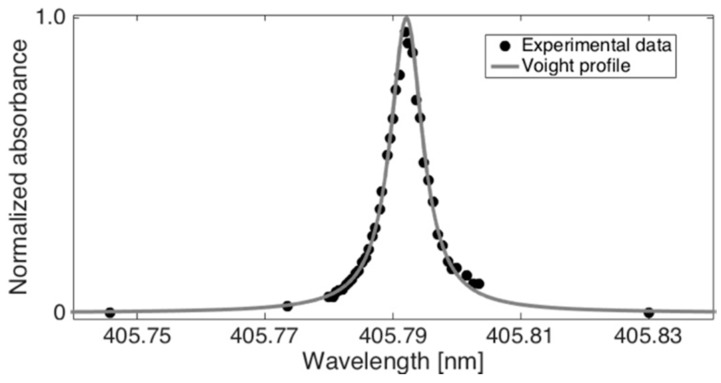
The absorption line of Pb* at 405.792 nm upon double photofragmentation of PbCl_2_. The profile was determined by measuring the absorbance as a function of the probe laser wavelength. The line represents a Voight profile obtained by fitting the experimental data (black dots).

Figure 3 shows the normalized absorption profile of the Pb* at 493 °C as a function of wavelength. Experimental values were obtained by tuning the wavelength of the probe beam across the Pb* absorption line at 405.789 nm with resolution of 0.5 pm. The experimental data was then fitted with a Voigt function to obtain the line shape and determine the absorption cross-section. The maximum of the experimental profile (405.792 nm) was about 11 pm shifted from the transition peak reported on the National Institute of Standards and Technology (NIST) atomic spectra database (405.7807 nm) [34]. The wavelength difference can be attributed to the pressure-induced shift in the high-temperature measurement cell. According to the Voigt fitting to the experimental data, the absorption cross-section at locking wavelength of 405.789 nm was σ_Pb_ = 1.9 × 10^−17^ m^2^.

PbCl_2_ absorption cross-section *σ_PbCl_*_2_ was determined experimentally by measuring the CPFAAS signal amplitude as a function of energy of the 355 nm pulses. The energy of the first fragmenting laser pulses at 355 nm was increased up to saturation of absorption of the probe signal [36]. The energy of the second laser pulse was kept constant for the measurements. The PbCl_2_ absorption cross section *σ_PbCl_*_2_ at 355 nm was determined to be 1.5 × 10^−21^ m^2^.

The kinetics of metal halide vapors, including PbCl_2_, have been extensively studied [10,35,37]. However, the UV absorption cross section of PbCl (*σ_PbCl_*) was not found in the literature. Thus, *σ_PbCl_* was determined experimentally by the means of molecular photodissociation and UV absorption spectroscopy. Broadband emission from a Deuterium–Halogen lamp (Ava-Light-DH(S)-BAL, Avantes) was used as a probe light for UV absorption of PbCl molecules in a 250–300 nm wavelength range. PbCl molecules were produced with photodissociation of PbCl_2_ molecules with 355 nm laser light. The UV light from the lamp was directed collinearly along the optical path of the 355 nm laser pulses through the PbCl_2_ vapor container. The transmitted light through the cell was directed into a spectrometer and detected by an intensified CCD camera (iStar, Andor) upon photodissociation. The exposure time for spectrum collection was 5 μs and it was triggered by the first fragmenting laser pulse. The collected PbCl absorption spectrum intensity *I* was compared to the lamp spectrum intensity *I*_0_

(11) $$\ln\left(\frac{I}{I_{0}}\right)\;=\;\frac{N_{PbCl}}{A}\sigma_{PbCl}$$

where *N_PbCl_* stands for the number of PbCl molecules after the dissociation that equals to the number of the absorbed photons with energy corresponding to wavelength of 355 nm [35]. The value of $\sigma_{PbCl}$ at 266 nm was measured at N_2_-O_2_ (96–4%) atmosphere resulting in 9.3 × 10^−22^ m^2^. The obtained cross-section values were in the same order of magnitude as the values for similar alkali halide vapors reported in the literature in the UV spectral range [10].

### 4.2. CPFAAS Signal Formation and Limit of Detection

**Figure 4:**
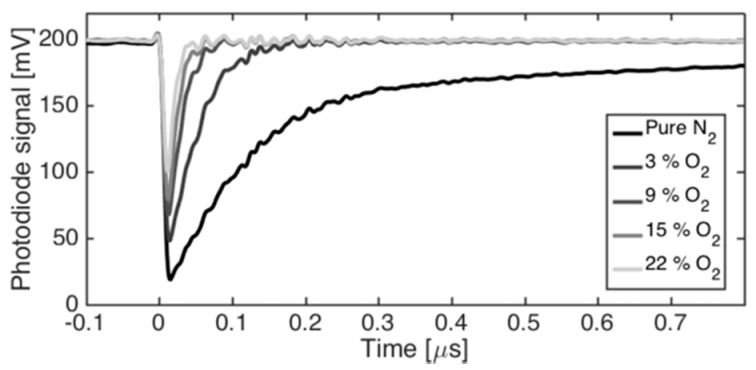
Examples of CPFAAS signal that shows the effect of O_2_ environment on the magnitude of a signal.

Examples of the temporal probe beam transmission curves after PbCl_2_ photodissociation are presented in Figure 4. The example curve is an average of 100 signals. The recorded photodetector signal shows a transmission decay of the transmitted probe beam at *t* = 0 that corresponds to the launch of the second fragmenting laser pulse. The relative depth of the temporal minimum in transmission corresponds to the absorbance *𝛼L_max_* after consecutive fragmenting laser pulses that were fired through the sample volume. The signal dip resulted from a sudden increase of Pb* atoms on the optical path inside the sample chamber and their ability to absorb light at the wavelength of the probe beam. Hence, the photon energy associated to the first fragmentation laser pulse with wavelength of 355 nm dissociated resonantly the PbCl_2_ molecules into PbCl in a ground state X as depicted in the diagram of Figure 2. The second absorption process of PbCl molecules was initiated by absorption of the second fragmenting laser pulse with wavelength of 266 nm that populated the electronic state B of the PbCl molecule. A predissociation process was achieved along the C curve into Pb* in the excited state 6p^2^ 3P_2_ [38]. Here, the transmission of the probe beam was reduced due to optical absorption into Pb*, producing the signal related to *𝛼L_max_*. The transmission signal decays to the base level after hundreds of nanoseconds following an exponential form, being in agreement with Equation (8). The signal of the transmitted beam had similar behavior to the CPFAAS signal observed earlier for KCl and KOH molecules [14,15,16].

Process gas environments, such as combustion flue gas, are reactive gas mixtures. Therefore, it is important to know how changes in gas composition affect the obtained CPFAAS signal due to its dependence on chemical reaction kinetics. O_2_ concentration was found to be the most critical factor on quantitative PbCl_2_ measurement with CPFAAS due to its reactivity and good availability on combustion gases. The lifetime of the PbCl molecules after the first fragmentation event was analyzed experimentally for N_2_ and N_2_-O_2_ (96–4%) environments by tuning the interpulse delay time, Δ*t*. The decay time of the PbCl radical concentration followed an exponential decay for both environments. At pure N_2_ atmosphere, the decay time was estimated to be 30 μs. For the N_2_-O_2_ atmosphere, the PbCl lifetime decreased to 14 μs. Thus, the Δ*t* played a major role on the signal formation in O_2_ environments since shorter Δ*t* led to stronger CPFAAS signal. In our experiments, Δ*t* was varying due to the accumulative effect of the laser jitters. Hence, to obtain credible quantitative data, Δ*t* was controlled to be below 1 μs by neglecting the signals recorded with interpulse delay exceeding this threshold value. Interpulse delay Δ*t* = 1 μs led to quantitative measurement uncertainty of 4% and 7% in N_2_ and in used N_2_-O_2_ atmospheres, respectively. In addition to PbCl lifetime, the presence of O_2_ affected the lifetime of Pb* by reducing it to nanosecond time scale. Figure 4 shows the combined effect of PbCl and Pb* lifetimes at different atmospheres on CPFAAS signal. Typically, the concentrations of major gases are fairly well known in industrial processes and, therefore, the effect of O_2_ concentration on quantitative PbCl_2_ monitoring can be mitigated by taking it into consideration in signal processing. However, this demonstrates how sequential CPFAAS can aid chemical kinetic research by providing direct measurement data on chemical reaction rates that previously had not been accessible. Chemical models and simulations rely often on computational kinetic values, especially in the case of radical intermediates. Providing feedback on such models is a topic of another research.

**Figure 5:**
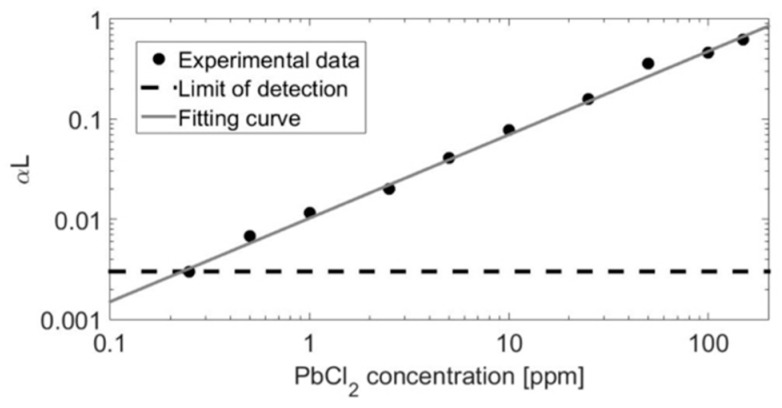
The CPFAAS signal as a function of PbCl_2_ concentration on a log-log scale. The PbCl_2_ concentration was varied by controlling the temperature of the sample container. The signal was produced by using 4 mJ and 0.1 mJ energies for the first and second fragmentation laser pulses with wavelengths of 355 nm and 266 nm, respectively. The calibration curve shows a dynamic range of 3 orders of magnitude and a limit of detection of 0.25 ppm.

The *𝛼L_max_* was evaluated as a function of the PbCl_2_ concentration in laboratory conditions to demonstrate the dynamic behavior of the measurement method and to obtain estimation for the limit of detection (LOD). The obtained concentration values were calculated using Equation (7). Figure 5 shows a log-log plot of the absorbance maximum, 𝛼L_max_, related to the PbCl_2_ concentration in a range from 0.3 ppm to 100 ppm. The PbCl_2_ concentration was varied by tuning the tube oven temperature and calculating the equilibrium vapor pressure of PbCl_2_ using HSC 5.1 database. The LOD for *𝛼L_max_* was determined as –ln(3σ/I_0_), where σ is the standard deviation of the photodiode signal before UV excitation. This corresponded to 0.25 ppm of PbCl_2_ with 4 mJ and 0.1 mJ pulse energies for the first and second fragmentation laser pulses with wavelengths of 355 nm and 266 nm, respectively, in a 0.6-m-long tube oven. However, lower LOD can be achieved by increasing laser pulse energies and extending the interaction length, as can be seen from Equations (7) and (10). The curve in Figure 5 shows good linearity across the three orders of magnitude of concentration over which the signal was analyzed. The linear behavior confirmed that the species’ UV absorption cross-sections were constant in the limits of measurement uncertainty within the temperatures from about 300 °C to 500 °C that were used to adjust the PbCl_2_ vapor in the measurement volume. Recent study on alkali compound UV absorption cross-sections has presented similar results that the metal-containing molecule UV absorption cross-sections showed only minor changes as a function of temperature [39]. This justifies that Equations (7) and (10) were used for quantitative PbCl_2_ monitoring in, e.g., dynamic combustion environment. The dynamic range of Figure 5 is suitable for the PbCl_2_ concentration expected on the combustion process of a full-scale power plant where the online performance of the method is further demonstrated [32,40].

### 4.3. Online PbCl_2_ Detection at a Full-Scale Power Plant

Successful detection of PbCl_2_ in gas phase was carried out on a full-scale power plant by a mobile and robust CPFAAS prototype device. The measurements were carried out in a 120 MW_th_ CFB boiler that uses recovered waste wood as the main fuel. PbCl_2_ concentrations were monitored close to the economizers (point A) and in the proximity of the superheater tubes (point B) where the flue gas temperatures provided by the boiler operator were 420 °C and 660 °C, respectively. At the measurement points A and B, the width of the flue gas channel was 10 m. During the plant measurements, the limestone usage was altered and its effect on PbCl_2_ concentration was monitored. Limestone (CaCO_3_) injection can be used to reduce SO_2_ emissions. CaCO_3_ decomposes in the furnace to calcium oxide (CaO) and CO_2_. CaO binds SO_2_ and forms calcium sulphate (CaSO_4_). However, sulphur capture typically increases the share of problematic chloride formation.

**Figure 6:**
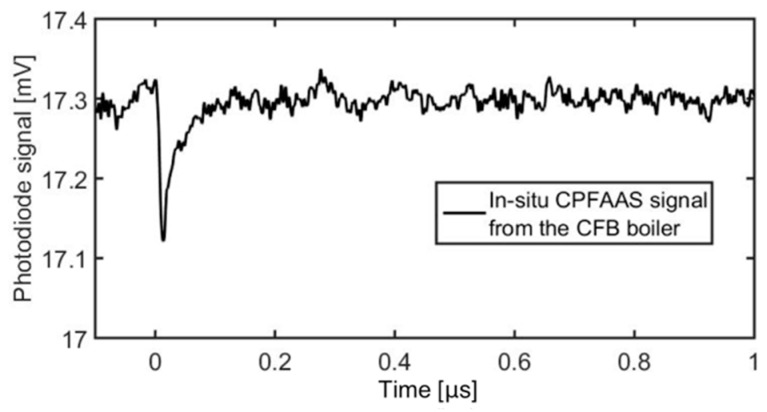
Example of a smoothed CPFAAS signal obtained directly from a full-scale power plant. The signal was obtained from 10-m-wide flue gas channel.

The prototype device consisted of a laser launch plate, a detector plate, a power supply unit, and a portable computer. The launch and detector plates were connected directly to the boiler wall to enable the laser diagnostics and to prevent airflow into the boiler. For all measurements, the output laser energies were kept on 20 mJ and 90 μJ for 355 nm and 266 nm laser pulses, respectively. The diameter of the beam cross-section area was 12 mm. Figure 6 shows the CPFAAS signal obtained in the plant measurements. The signal was an average of 1000 individual CPFAAS signals and it was smoothed with a moving average window of 25 points. The measurement conditions and the main results regarding the PbCl_2_ detection are summarized in Table 1. Concentration values were calculated with Equation (10). Due to the longer interaction length and higher laser pulse energies than those used in laboratory experiments, the LOD was substantially lower than the LOD demonstrated in a laboratory-scale reactor. The LOD for PbCl_2_ in full-scale boiler experiments was estimated using 3σ condition to be 0.01 ppm. For quantitative analysis, the average signal was estimated considering only the signals with interpulse delay of Δt<2 μs. The tolerance in Δ*t* was increased to shorten the acquisition time as also signals without corresponding fragmenting laser pulse energy reading at the detector plate were neglected. Extension of Δ*t* increased the measurement uncertainty arising from the computation method assuming Δ*t* = 0 to 14%. A large variation to the optical transmission of the fragmenting laser beams was induced due to the local temperature fluctuations in the boiler. Therefore, the PbCl_2_ concentrations reported in Table 1 were calculated from a signal that was an average of 1000 accumulated individual signals. Here, the acquisition time was in the order of 2 min since the repetition rate of the laser output was 10 Hz. Thus, the duty-cycle of the measurements was adequate for online monitoring of the boiler operation.

The measured signal was weighted to the first few meters of the laser propagation path due to strong scattering and absorption of the fragmenting laser pulses. The results in Table 1 were calculated assuming even distribution of PbCl_2_ along the measurement path. Therefore, the measured absolute concentration value may be erroneous if PbCl_2_ was not distributed evenly along the measurement path. However, the measured values can be used as indicators of relative change in PbCl_2_ concentration. It was found that the use of limestone in feedstock increased the PbCl_2_ concentration in flue gas at measured points A and B with 29% and 81%, respectively. This confirms that sulphur capture increased the formation of PbCl_2_. Nevertheless, the determined PbCl_2_ concentration values were substantially lower than was expected based on thermodynamic equilibrium calculations for similar fuels and combustion conditions [32,40]. There are several possible reasons for the discrepancy, one being that chemical equilibrium may not be reached in the boiler given the short residence time, relatively low temperature, and heterogenous fuel particle distribution. Also, there might have been shortcomings in the thermodynamic databases used. Moreover, the chemical compositions of the fuels used in the equilibrium calculations were not identical to the fuels used during the measurements, hence, the comparison is only indicative. In addition, as the measurements in laboratory conditions demonstrated, lead reaction dynamics are heavily affected by the surrounding atmosphere. This has a major impact on reaction rates and gas-phase radical lifetimes. As the chemical reaction kinetics for lead species, to the authors’ knowledge, are not sufficiently well known, the simulation values can only be considered as approximate values. CPFAAS technique could be further utilized to study the lead reaction kinetics to improve the chemical kinetics databases and simulations [41]. Regardless, the results show the potential and applicability of the CPFAAS method to measure PbCl_2_ in the full-scale power plant environment. The results obtained in the laboratory and in the full-scale studies also imply that further investigations on lead reaction kinetics are required to improve both the accuracy of the CPFAAS measurement method and the chemical simulations.

## 5. Conclusions

This work is the first demonstration of sequential CPFAAS measurement of a triatomic molecule. Sequential double dissociation process enables detection of, for example, PbCl_2_ in laboratory conditions and, furthermore, in a full-scale power plant boiler. For quantitative determination of PbCl_2_ concentration in gas phase, the UV absorption cross-sections for PbCl_2_, PbCl, and Pb* were determined to be *σ_PbCl_*_2_ = 1.5 × 10^−21^ m^2^, *σ_PbCl_* = 9.3 × 10^−22^ m^2^, and σ_Pb_ = 1.9 × 10^−17^ m^2^, at wavelengths of 355 nm, 266 nm, and 405.789 nm, respectively. The determined absorption cross-sections enable calibration free quantitative monitoring of PbCl_2_ in an arbitrary environment. In a laboratory-scale tube furnace, the LOD for PbCl_2_ was determined to be 0.25 ppm. However, the LOD can be improved with higher laser pulse energies or with longer interaction length. Previously, CPFAAS was utilized for chemical kinetic studies and reaction environment monitoring using the CPFAAS signal recovery rates from KCl and KOH [41,42]. The laboratory-scale experiments showed that the sequential CPFAAS has potential to be used for similar kinetic studies of triatomic molecules and radicals that are formed as intermediate fragments. This feature has a lot of application potential for providing feedback on chemical reaction models and reaction kinetic databases. The obtained results from the flue gas channel of the demolition-wood-fired 120 MW_th_ CFB boiler are a powerful demonstration of the method’s applicability to in situ monitoring of metal-containing triatomic species in process chemistry. The results showed that the use of limestone with the feedstock may increase the PbCl_2_ concentration in gas phase by up to 81% and that the obtained concentration values are substantially lower when compared to thermodynamic equilibrium calculations. This type of information on gas-phase chemistry will improve the chemical simulations of industrial processes by providing crucial comparison for their numerical values and, on the other hand, providing new chemical kinetic information for the simulation parameters.

## Figures and Tables

**Table 1:** Measurement parameters and main results from PbCl_2_ detection through a 10-m-wide flue gas passage using the CPFAAS prototype.

Location	Limestone Addition	Transmittance (%)		Probe I/I_0_	PbCl_2_ (ppm)
		355 nm	266 nm		
A (420 °C)	No	5.5	1.8	0.99	0.07 ± 0.01
	Yes	2.0	1.2	0.99	0.09 ± 0.02
B (660 °C)	No	8.3	0.8	0.96	0.43 ± 0.07
	Yes	4.7	0.3	0.94	0.78 ± 0.11
